# Urokinase-loaded cyclic RGD-decorated liposome targeted therapy for *in-situ* thrombus of pulmonary arteriole of pulmonary hypertension

**DOI:** 10.3389/fbioe.2022.1038829

**Published:** 2022-10-17

**Authors:** Jingtao Li, Xiaofeng Zhang, Yingying Mo, Tongtong Huang, Huaqing Rao, Zhenyuan Tan, Liuliu Huang, Decai Zeng, Chunlan Jiang, Yanfen Zhong, Yongzhi Cai, Binbin Liang, Ji Wu

**Affiliations:** ^1^ Department of Ultrasound, The First Affiliated Hospital of Guangxi Medical University, Nanning, China; ^2^ Pharmaceutical College, Guangxi Medical University, Nanning, China; ^3^ Department of Cardiothoracic Surgery, The First Affiliated Hospital of Guangxi Medical University, Nanning, China

**Keywords:** cyclic RGD peptide, liposome, *in-situ* thrombus, targeted thrombolysis, pulmonary hypertension, right ventricular function

## Abstract

**Backgroud:** *In-situ* thrombosis is a significant pathophysiological basis for the development of pulmonary hypertension (PH). However, thrombolytic therapy for *in-situ* thrombus in PH was often hampered by the apparent side effects and the low bioavailability of common thrombolytic medications. Nanoscale cyclic RGD (cRGD)-decorated liposomes have received much attention thanks to their thrombus-targeting and biodegradability properties. As a result, we synthesized urokinase-loaded cRGD-decorated liposome (UK-cRGD-Liposome) for therapy of *in-situ* thrombosis as an exploration of pulmonary hypertensive novel therapeutic approaches.

**Purpose:** To evaluate the utilize of UK-cRGD-Liposome for targeted thrombolysis of *in-situ* thrombus in PH and to explore the potential mechanisms of *in-situ* thrombus involved in the development of PH.

**Methods:** UK-cRGD-Liposome nanoscale drug delivery system was prepared using combined methods of thin-film hydration and sonication. Induced PH *via* subcutaneous injection of monocrotaline (MCT). Fibrin staining (modified MSB method) was applied to detect the number of vessels within-situ thrombi in PH. Echocardiography, hematoxylin-eosin (H & E) staining, and Masson’s trichrome staining were used to analyze right ventricular (RV) function, pulmonary vascular remodeling, as well as RV remodeling.

**Results:** The number of vessels with *in-situ* thrombi revealed that UK-cRGD-Liposome could actively target urokinase to *in-situ* thrombi and release its payload in a controlled manner in the *in vivo* environment, thereby enhancing the thrombolytic effect of urokinase. Pulmonary artery hemodynamics and echocardiography indicated a dramatical decrease in pulmonary artery pressure and a significant improvement in RV function post targeted thrombolytic therapy. Moreover, pulmonary vascular remodeling and RV remodeling were significantly restricted post targeted thrombolytic therapy.

**Conclusion:** UK-cRGD-Liposome can restrict the progression of PH and improve RV function by targeting the dissolution of pulmonary hypertensive *in-situ* thrombi, which may provide promising therapeutic approaches for PH.

## Introduction

**FIGURE 1:**
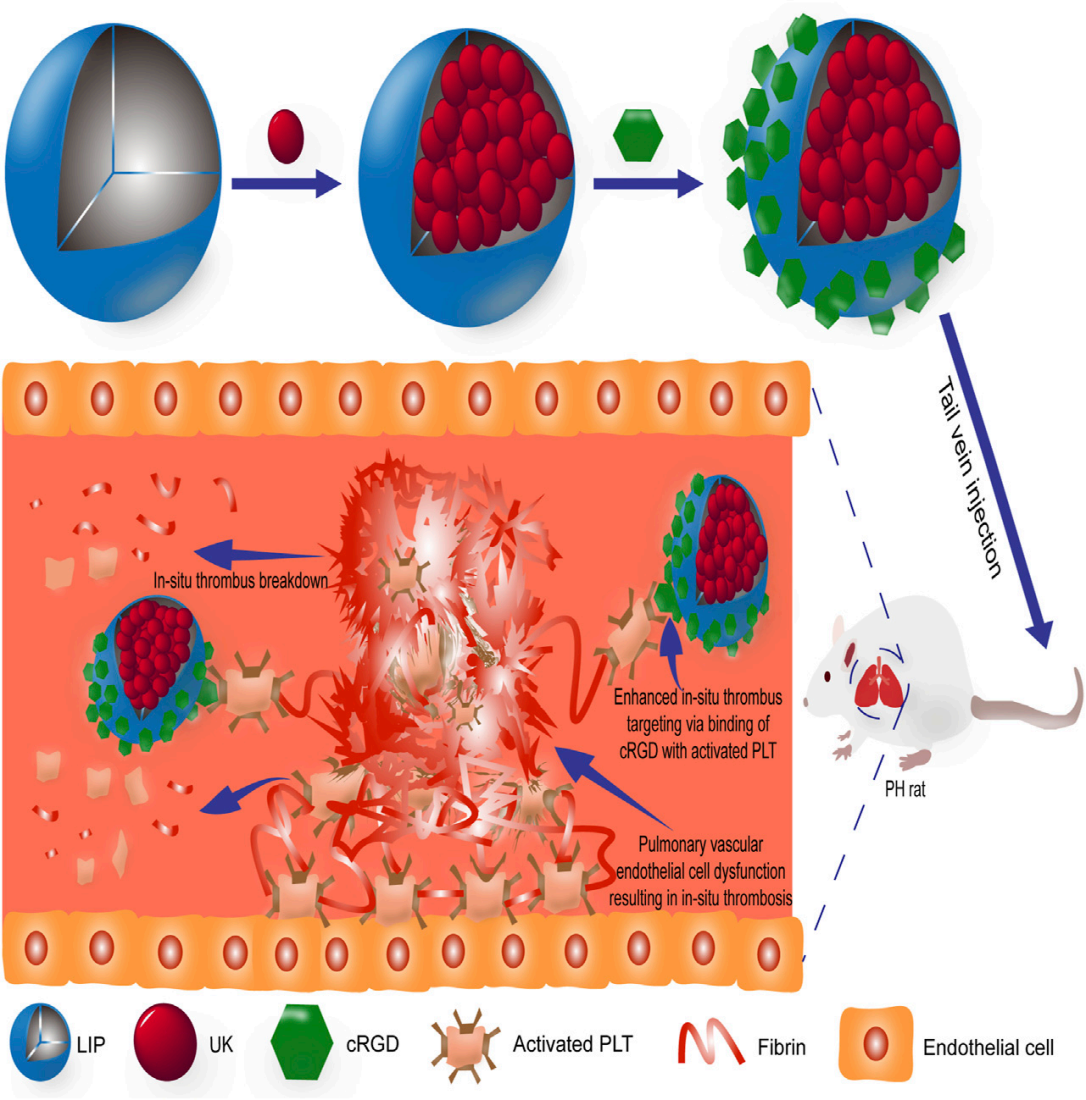
Schematic illustration of the mechanism of targeted therapy of *in-situ* thrombus in PH with UK-cRGD-Liposome nanoparticles. Abbreviations: PH, pulmonary hypertension; cRGD, Cyclic RGD; UK, urokinase; PLT, platelet.

PH is a life-threatening chronic disease characterized by pulmonary vasoconstriction, pulmonary vascular remodeling, and *in-situ* thrombosis that induces a progressive increase in pulmonary vascular resistance, ultimately leading to right heart failure and death (Vonk-Noordegraaf et al., 2013; Paulin et al., 2015). Currently, pharmacological therapies for PH focus on the imbalance between vasoconstriction and vasodilation, which mainly includes drugs such as prostacyclin receptor agonists, endothelin receptor antagonists (ERAs), and phosphodiesterase type 5 inhibitors (PDE-5is) (Velayati et al., 2016; Sommer et al., 2021). Although such therapies have improved the vascular function of the patient, the survival rate is still unsatisfactory (Boucly et al., 2017; Jalce and Guignabert, 2020). Clinical therapies for *in-situ* thrombosis of PH are mainly concentrated on anticoagulant therapy, but forceful clinical evidence to verify its usefulness and efficacy is lacked (Olschewski and Rich, 2018; Cullivan et al., 2021; Rawal et al., 2021). Previous studies have reported that *in-situ* thrombi of pulmonary arterioles in idiopathic pulmonary hypertension and experimental pulmonary hypertension were micro-thrombotic composed of aggregated activated platelets and deposited fibrin (Figure 1) (Fan et al., 2019). Since micro-thrombosis is very tiny (micron scale) and relatively stable, conventional thrombolytic therapy has little effect on its treatment. In addition, *in-situ* thrombosis of pulmonary arteriole could lead to more severe PH (White et al., 2007; Fernandez et al., 2012). Therefore, it is of great importance to develop a novel targeted therapy method for curing pulmonary arteriole *in-situ* thrombus in PH.

Thrombolytic therapy is so far the main method used clinically to dissolve blood clots and restore vessel patency (Vaidya et al., 2012; Zhou et al., 2021). A variety of thrombolytic drugs, such as urokinase, streptokinase, and tissue-type fibrinogen activators, which can convert fibrinolytic zymogen to fibrinolytic enzymes and thus degrade the thrombus backbone fibrin (Liu et al., 2007; Hu et al., 2019), these thrombolytic drugs for a short plasma clearance half-life and the lack of thrombus targeting, large drug doses are required for effective thrombolysis. However, heavy use of thrombolytic drugs can over-activate the systemic fibrinolytic system, resulting in severe hemorrhagic side effects (Koudelka et al., 2016; Altaf et al., 2021). Furthermore, incomplete thrombolysis could lead to embolism of the distal vascular vessels. Consequently, how to improve thrombolytic drugs’ bioavailability, plasma clearance half-life, and at the same time reduce systemic side effects are key to thrombolytic therapy.

Liposomes have numerous advantages, such as good biocompatibility, biodegradability, low cytotoxicity, and easy surface modification (Akbarzadeh et al., 2013; Khan et al., 2020). Meanwhile, PEGylated liposome is an ideal drug carrier due to inhibiting the clearance of the mononuclear macrophage system and improving liposome stability and *in vivo* circulation time (Sercombe et al., 2015). Integrin αIIbβ3 (also called GPIIb/IIIa) is a glycoprotein on the surface of activated platelets that promotes platelet aggregation and thrombosis (Guo et al., 2015). GPIIb/IIIa on the surface of activated platelets could be specifically recognized by RGD peptides (Pawlowski et al., 2017). cRGD peptides have higher affinity and enzymatic stability with GPIIb/IIIa glycoproteins than linear RGD peptides, which are ideal ligands for targeting the thrombus where activated platelets are gathering (Huang et al., 2008). Previous studies by Zhang et al. (2018), as well as our research group (Rao, 2020; Rao et al., 2021), have demonstrated that UK-cRGD-Liposome has the advantages of targeting activated platelets (Figure 1), improving the plasma clearance half-life of thrombolytic drugs, and reducing hemorrhagic side effects.

Integrin ανβ3, which is highly expressed in a variety of tumor cells and tumor vascular endothelial cells, could be specifically bound with RGD peptide (Pan et al., 2019). Several *in vivo* targeting results have also showed that RGD peptide was able to target tumor cells of many cancer types, including breast, colon, gastric, and lung cancers, etc (Garcia Ribeiro et al., 2019; Chen et al., 2021; Liu et al., 2022). In contrast, UK-cRGD-Liposome has only been studied *in vivo* in target-seeking studies of acutely formed micro thrombus (Zhang et al., 2018). Its *in vivo* target-seeking studies of chronically formed pulmonary arteriole *in-situ* thrombus in PH are still blank, and whether its treatment effect is better than conventional thrombolytic drugs or anticoagulant in chronically formed pulmonary arteriole *in-situ* thrombus is also still unclear.

Thus, the goal of this study was to establish an animal model of PH in order to explore the thrombolytic efficacy of UK-cRGD-Liposome in pulmonary arteriole *in-situ* thrombus in PH as well as the relationship between pulmonary arteriole *in-situ* thrombus and the process of PH, which could lead to new ideas and basic research evidence for the targeted therapy of PH.

## Material and methods

### Material and animals

Monocrotaline (MCT) and sodium pentobarbital were purchased from Sigma, USA. Beijing Solarbio Science & Technology Co. supplied 1,2-dipalmitoyl-sn-glycero-3-hosphocholine (DPPC), cholesterol, fibrin staining solution, and Masson‘s trichrome staining kits. 1,2-distearoyl-sn-phosphoethanolamine-N-[methoxy (polyethylene glycol)-2000] (DSPE-mPEG-2000) and 1,2-distearoyl-sn-phosphoethanolamine-N-[methoxy (polyethylene glycol)-2000]-cRGD (DSPE-mPEG2000-cRGD) were synthesized by Hunan Huateng Pharmaceutical Co. Urokinase for injection (250,000U) was acquired from the First Affiliated Hospital of Guangxi Medical University’s central pharmacy.

Eighty-five pathogen-free inbred 8-weeks-old male Sprague-Dawley (SD) rats were purchased from the Animal Experiment Center of Guangxi Medical University (certificate number: SCXK GUI 2020-0003).

### Preparation of UK-cRGD-liposome

UK-cRGD-Liposome was prepared using integrated methods of thin-film hydration and sonication (Zhang et al., 2018; Rao, 2020). DPPC, Cholesterol, DSPE-mPEG2000, and DSPE-mPEG2000-cRGD (ratio of 12 mg: 6 mg: 2 mg: 1 mg) were dissolved in chloroform. The solution dissolved all the solutes in a water bath sonicator and was preliminarily dried using a rotary evaporator (Yarong Biochemical Instrument Factory, China) to form a thin film of cRGD liposome. The cRGD liposome film was then dried in a vacuum dryer overnight to evaporate the organic solvent. The next day, lysis of cRGD liposome film membranes with the phosphate buffer solution (PBS) of urokinase (5 ml, 50,000 U/ml) combined with a water bath sonicator. The ultrasonic cell disruption instrument (4°C, 5/5 s, on/off, 320w) was used to reduce the size of UK-cRGD-Liposome for 10 min and then centrifuge for 5 min (4°C, 3,000 rpm) to produce a limpid suspension of UK-cRGD-Liposome. The suspension was transferred to a dialysis bag (molecular weight cut off 300 kDa) and dialyzed with 2 L of PBS buffer for 6 h to remove unencapsulated urokinase.

DPPC, Cholesterol, and DSPE-mPEG2000 (ratio of 12 mg: 6 mg: 3 mg) were dissolved in chloroform, and the remaining steps were performed as above to obtain urokinase-loaded liposome (UK-Liposome).

### Animal grouping and administration

**FIGURE 2:**
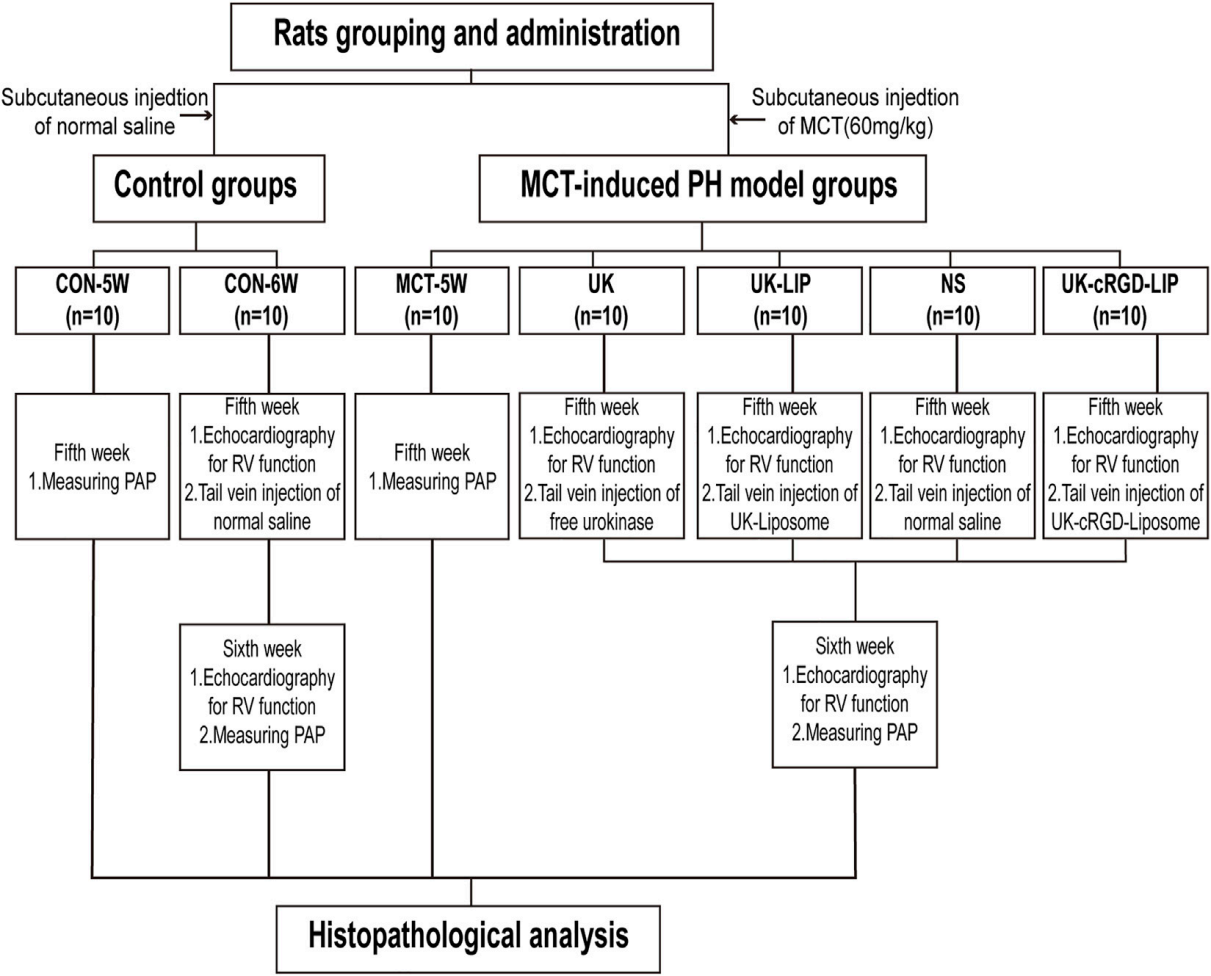
Flowchart of grouping and administration of rats in each group. Abbreviations: MCT, monocrotaline; RV, right ventricular; PAP, pulmonary artery pressure; UK-Liposome, urokinase-loaded liposome; UK-cRGD-Liposome, urokinase-loaded cycle RGD-decorated liposome.

The temperature of the rat rearing environment was regulated at 20 ± 2°C, the humidity was kept at 60 ± 5%, and a 12-h:12-h light-dark cycle was used. Previous studies have shown that MCT-induced PH rats develop *in-situ* thrombosis at week three (Fan et al., 2019) and right heart failure at week five or six (Paulin et al., 2015; Deng et al., 2017). Therefore, this study proposed administering thrombolytic therapy at week five after MCT injection and monitoring it for 1 week. Seventy SD rats were randomly divided to MCT (*n* = 50) or control (*n* = 20) groups (Figure 2). The MCT group received a single subcutaneous injection of MCT (60 mg/kg), while the control group received an equivalent volume of saline. Each group of rats was further subdivided according to duration after MCT injection (5, 6 weeks) and thrombolytic treatment modality (normal saline, free urokinase, UK-Liposome, UK-cRGD-Liposome). These subgroups were referred to as MCT-5W (*n* = 10), NS (*n* = 10), UK (*n* = 10), UK-LIP (*n* = 10), UK-cRGD-LIP (*n* = 10), and CON-5W (*n* = 10), CON-6W (*n* = 10), respectively. In the fifth week after MCT injection, rats in the MCT-5W and CON-5W groups measured pulmonary artery pressure *via* echocardiography-guided transthoracic puncture. Following invasive hemodynamic analysis, the heart and lung tissues of rats were pathologically examined to determine the degree of pulmonary vascular remodeling and RV remodeling and whether *in-situ* thrombi in pulmonary arterioles were formed. Five weeks after MCT injection, echocardiography was performed on rats in the CON-6W, NS, UK, UK-LIP, and UK-cRGD-LIP groups. Then, rats in the NS, UK (100 U/g), UK-LIP (100 U/g), and UK-cRGD-LIP (100 U/g) groups were injected with appropriate amounts of thrombolytic medicines *via* the tail vein. One week after thrombolytic therapy, echocardiography was performed again in rats in the CON-6W, NS, UK, UK-LIP, and UK-cRGD-LIP groups. The heart and lung tissues were removed for pathological examination after an echocardiography-guided transthoracic puncture to evaluate pulmonary artery pressure.

Fifteen SD rats were randomly divided into three groups: a blank control group (Control, *n* = 5); an UK-cRGD-Liposome group (*n* = 5); and an UK-Liposome (*n* = 5). The Control group received saline treatment, and the UK-cRGD-Liposome and UK-Liposome groups were administered with appropriate medicines injected *via* the tail vein. Pathological evaluation of heart, liver, spleen, lung, and kidney tissues was performed 1 week following treatment.

### Echocardiography

The rats were anesthetized *via* intraperitoneal administration of 2% sodium pentobarbital (50 mg/kg) and subjected to transthoracic echocardiography using a GE Vivid E95 ultrasound diagnostic machine (General Electric Company, USA) with an 18.0 MHz ultrasound probe (L8-18I-D). RV anterior wall thickness (RVAWT) was measured in a left ventricular long-axis view. Pulmonary artery acceleration time (PAAT) was assessed by Pulse-wave Doppler in short-axis views of the parasternal. RV end-diastolic transverse diameter (RVEDD), RV end-diastolic volume (RVEDV), RV end-systolic volume (RVESV), RV end-diastolic area (RVEDA), and RV end-systolic area (RVESA) were measured in apical four-chamber views. RV ejection fraction (RVEF%) = (RVEDV-RVESV)/RVEDV×100%, RV area change fraction (RVFAC%) = (RVEDA-RVESA)/RVEDA×100%. Tricuspid annular plane systolic excursion (TAPSE) was measured by M-mode echocardiography. The tricuspid annular movement was detected using tissue Doppler imaging in the apical cardiac four-chamber view, then to obtain isovolumic contraction time (ICT), isovolumic relaxation time (IRT), and ejection time (ET). RV myocardial work index (RV Tei index) = (ICT + IRT)/ET. RVEF, RVFAC, TAPSE, and RV Tei index were used to evaluate RV systolic function. RVEDD and RVAWT were used to assess right ventricular morphology. All examinations were performed by a sonographer who was blinded to the groups, and each parameter was measured three times and averaged.

### Invasive pulmonary artery hemodynamic measurements

**FIGURE 3:**
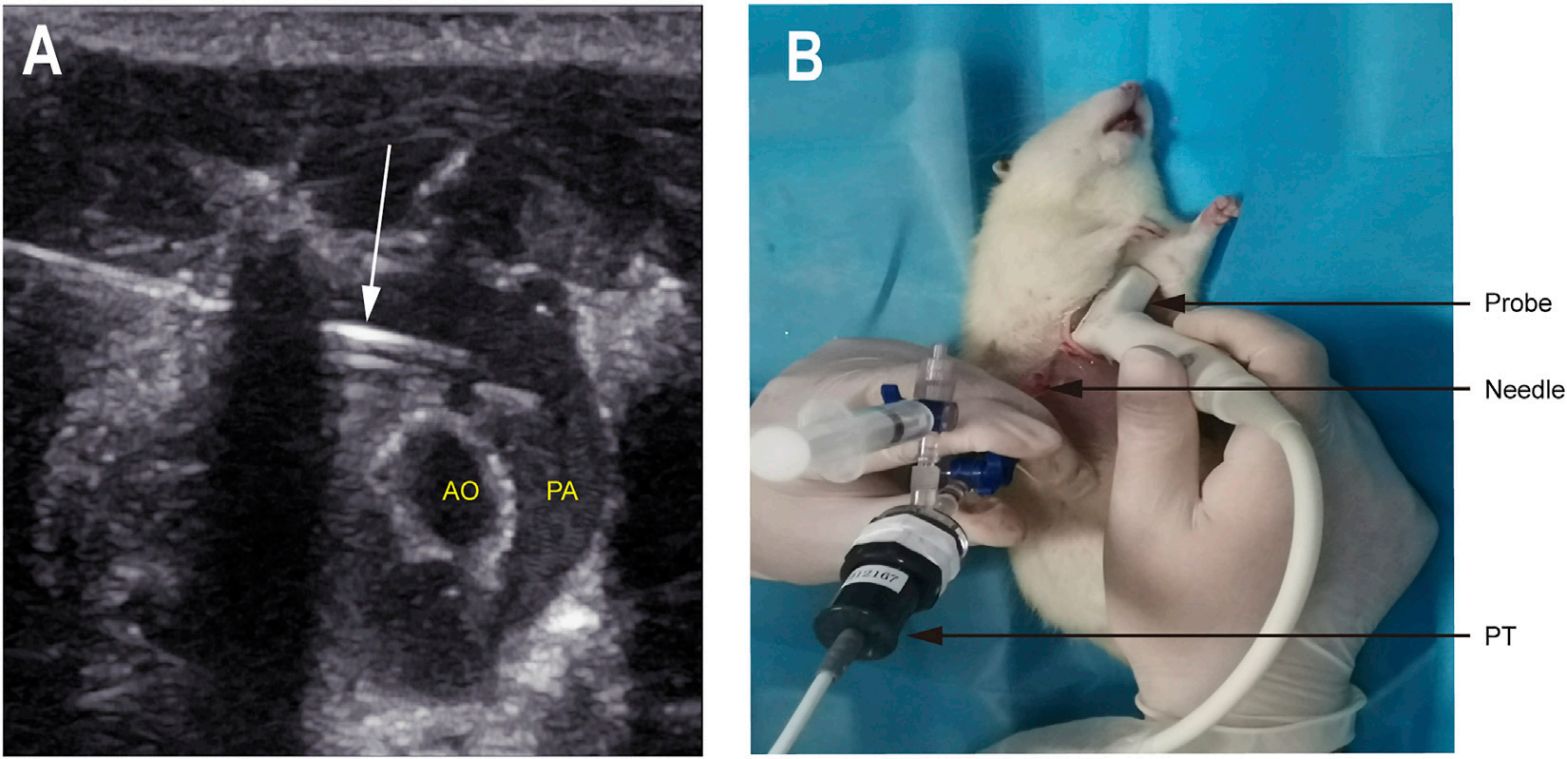
Echocardiography guided transthoracic puncture measurement of pulmonary artery pressure. **(A)** The puncture needle tip (white arrow) was located in the PA. **(B)** The actual operation of PA puncture by echocardiography was guided. Abbreviations: PA, pulmonary artery; AO, aorta; PT, pressure transducer.

The puncture needle was connected to the pressure transducer (PT) through a tee tube, and a syringe was connected to the tee tube and injected with heparin saline (100 U/mL) to maintain pressure (Figure 3B). Under the guidance of real-time echocardiography, the surgeon carefully inserted the puncture needle into the right ventricular outflow tract in the parasternal short-axis view (Figure 3A). After the right ventricular pressure curve appeared on the BL-420 F Biosignal Acquisition and Analysis System (Chengdu Taimeng Software Co., Ltd.), the puncture needle continued to be slowly advanced to the main pulmonary artery and recorded the pulmonary artery pressure curve.

### Histopathological analysis

After completion of pulmonary artery pressure detection, the rats were euthanized with an overdose of sodium pentobarbital. The thoracic cavity was opened quickly, and saline was injected steadily and slowly from the RV outflow tract (at the puncture hole) to flush the Cardiac and lung tissue, which could prevent excess blood and blood cells from remaining in the cardiac cavity or pulmonary vessels. Right ventricular hypertrophy index (RVHI) was calculated using the formula: RVHI (%) = RV/(LV + IVS) × 100%. RV hypertrophy was evaluated by RVHI (Yang et al., 2014). RV myocardial tissue and lung tissue were fixed with 4% paraformaldehyde for 48 h, routinely dehydrated, paraffin-embedded, and sectioned (5 μm).

Hematoxylin-eosin (H & E) staining: H & E staining was performed on lung tissue sections. Images were captured using an EVOS new inverted microscope imaging system (Life Technologies, United States). Using ImageJ image analysis software to measured vertical vessel external diameter (ED), vertical vessel inner diameter (ID), vessel total area (TA), and vessel lumen area (LA). Pulmonary arteriole morphology was assessed using the quantitative analysis methods proposed by Price et al. (2011), and Wu et al. (2016). with the calculation formulas pulmonary arteriole wall thickness percentage (wt%) = (ED-ID)/ED X 100% and pulmonary arteriole wall area percentage (WA%) = (TA-LA)/TA X 100%. Ten pulmonary arterioles (20–100 μm in diameter) were randomly selected for measurement in each section.

Fibrin staining (modified MSB method): Fibrin staining was performed on lung tissue sections according to the kit instructions. After staining, fibrin in the vessel was red, and red blood cells were yellow. 100 pulmonary arterioles (20–100 μm in diameter) were randomly observed and tallied as the number of vessels with residual *in-situ* thrombus for each rat in the MCT group. Right ventricular myocardial fibrosis was observed by Masson‘s trichrome staining. After staining, the collagen fibers were blue, and the myocardial tissue was red. Collagen volume fraction (CVF) was obtained by measuring the percentage of blue collagen fibers in myocardial tissues using ImageJ image analysis software (Guo et al., 2014). Ten slices of RV myocardial tissue were taken from each group of rats for evaluation.

### Statistical analysis

Statistical Product and Service Solutions software Version 26.0 (SPSS, IBM, USA) was used for statistical analysis. All values were expressed as Mean ± SD (standard deviation). An unpaired Student’s t-test was used to compare the data of two groups. For comparisons among multiple-group, the data with normal distribution and homogeneity of variance were compared by one-way ANOVA, otherwise by Kruskal–Wallis test. *p* < 0.05 was considered statistically significant.

## Results

### MCT-induced PH

MCT rats showed clinical signs of right heart failure such as shortness of breath, pleural fluid, ascites, and decreased physical activity in the fifth week after MCT injection, which was consistent with previously reported (Paulin et al., 2015; Deng et al., 2017). During the induction period, the mortality rates of rats in the MCT-5W, NS, UK, UK-LIP, and UK-cRGD-LIP groups were 10% (1/10), 20% (2/10), 20% (2/10), 20% (2/10), and 10% (1/10), respectively. In contrast, rats in the CON-5W and CON-6W groups all survived. No rats died in the NS, UK, UK-LIP, and UK-cRGD-LIP groups during the thrombolytic therapy period.

**FIGURE 4:**
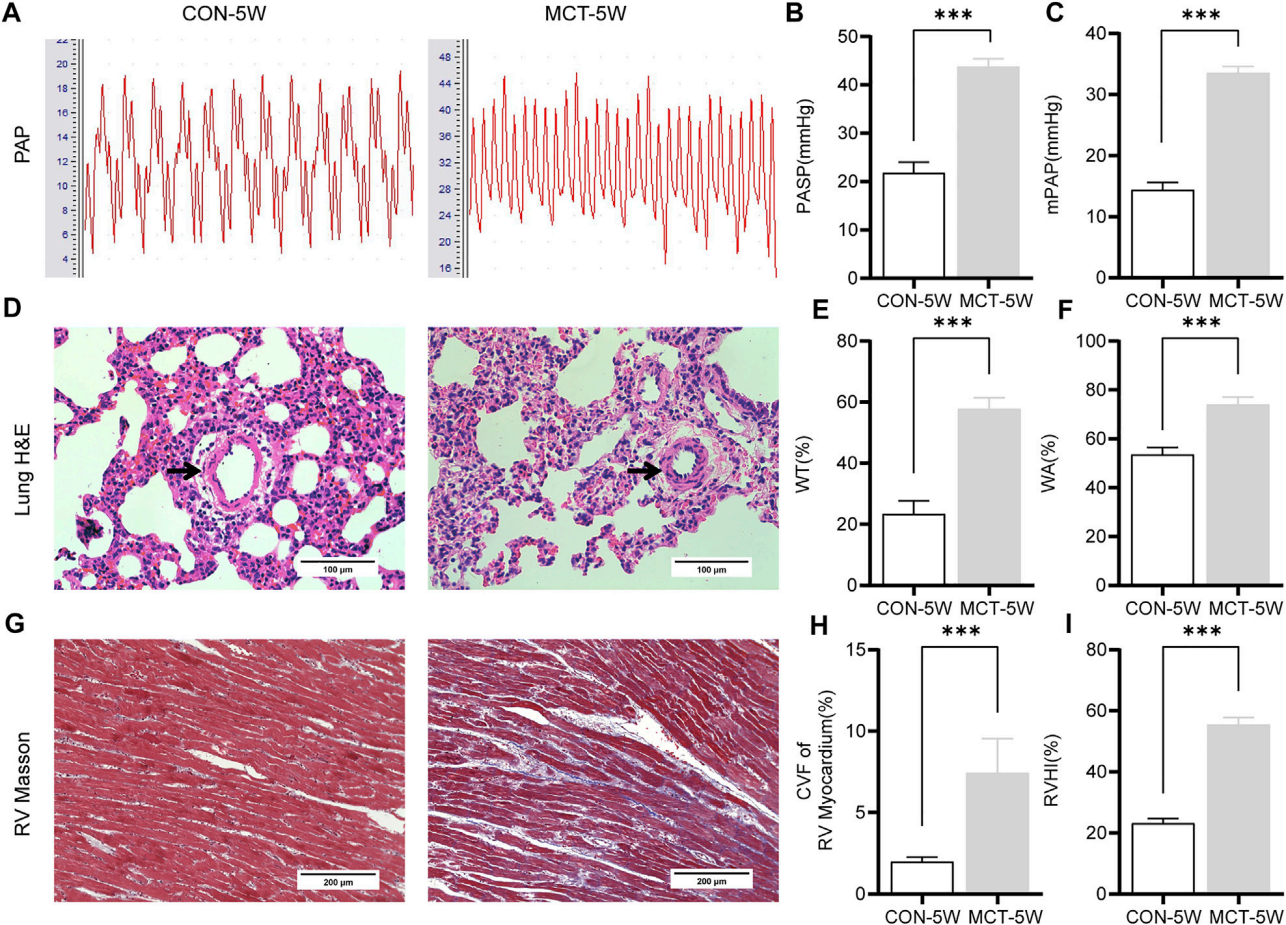
MCT-induced PH in rats. **(A)** Representative pulmonary artery pressure wavaforms obtained from CON-5W and MCT-5W rats. **(B)** Pulmonary artery systolic pressure was measured in CON-5W and MCT-5W rats. **(C)** Mean pulmonary artery pressure was measured in CON-5W and MCT-5W rats. **(D)** Representative images (×400 original magnification) of H & E staining of lung tissues from CON-5W and MCT-5W rats. Black arrow points to pulmonary arteriole (20–100 μm in diameter). **(E)** Quantitative analysis of vascular wall thickness **(F)** Quantitative analysis of vascular wall area **(G)** Representative images (×200 original magnification) of Masson’s trichrome staining of RV tissues from CON-5W and MCT-5W rats. Red indicates muscle fibers; blue indicates collagen fibers. **(H)** Quantitative analysis of fibrotic area **(I)** Quantitative analysis of right ventricular hypertrophy index. Values are presented as Mean ± SD. Data (CON-5W, *n* = 10; MCT-5W, *n* = 9) were analyzed by unpaired Student’s t-test. ***p < 0.001. Abbreviations: PASP, pulmonary artery systolic pressure; mPAP, mean pulmonary artery pressure; H & E, hematoxylin-eosin; WT, pulmonary arteriole wall thickness; WA, pulmonary arteriole wall area; CVF, collagen volume fraction; RVHI, right ventricular hypertrophy index.

Compared with the CON-5W group, both pulmonary artery systolic pressure (PASP) (Figures 4A,B) and mean pulmonary artery pressure (mPAP) (Figures 4A,C) were significantly higher in the MCT-5W group rats. H& E staining of lung tissue sections revealed significant pulmonary vascular remodeling in the MCT-5W rats (Figures 4D–F). Furthermore, RV myocardial fibrosis (Figures 4G,H) and RV remodeling were also significantly aggravated (Figure 4I). These histopathological results further validated the pulmonary artery hemodynamic findings. In addition, fibrin staining (modified MSB method) of lung tissue sections also confirmed the presence of *in situ* thrombus dominated by fibrin deposition in pulmonary arteriole in the MCT-5W group of rats (Figure 4).

### UK-cRGD-liposome for *in-situ* thrombus

**FIGURE 5:**
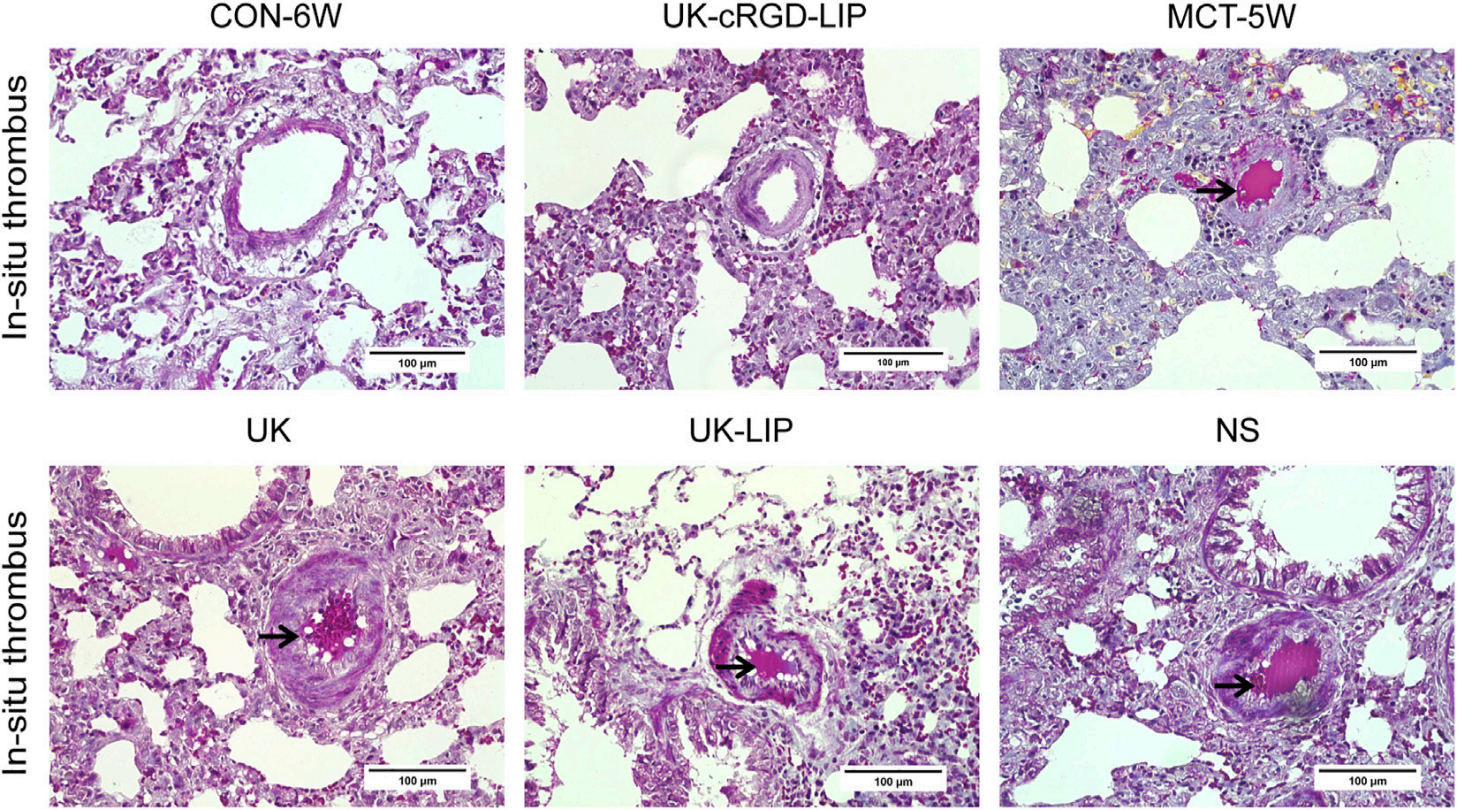
Representative images (×400 original magnification) of fibrin staining (modified MSB method) of lung tissues from CON-6W, MCT-5W, UK, UK-LIP, NS, and UK-cRGD-LIP rats. Red indicates fibrin deposition; yellow indicates erythrocyte accumulation. Black arrow points to intravascular *in-situ* thrombus.

UK-cRGD-Liposome could target dissolve of pulmonary arteriole *in-situ* thrombus in PH. As known by fibrin staining (modified MSB method) of lung tissue sections, the number of vessels with *in-situ* thrombi in rats in the UK-cRGD-LIP group was clearly less than that in the NS, UK, and UK-LIP groups (Table 1 and Figure 5). In addition, we found that the number of vessels with *in-situ* thrombi was further increased in the UK and UK-LIP groups compared with the MCT-5W group, whereas it was markedly decreased in the UK-cRGD-LIP group (Table 1). The above data suggest that UK-cRGD-Liposome improves the thrombolytic efficacy of urokinase by actively targeting urokinase delivery to *in-situ* thrombus.

**TABLE 1:** The number of vessels with residual in-situ thrombus.

Group	N	Vessels
MCT-5W	9	30.6 ± 2.3
UK	8	36.4 ± 3.2**
UK-LIP	8	35.5 ± 3.0*
NS	8	41.5 ± 4.1**^#^
UK-cRGD-LIP	9	19.7 ± 3.2**^##▲▲^

Notes: 100 pulmonary arterioles (20–100 μm in diameter) were randomly observed. Values were presented as Mean ± SD. Data were analyzed by one-way ANOVA. ^*^ *p* < 0.05 and ^**^ *p* < 0.01 vs. MCT-5W. ^#^ *p* < 0.05 and ##*p* < 0.01 vs. UK or UK-LIP; ^▲▲^ *p* < 0.01 vs. NS.

### Pulmonary vascular remodeling, RV remodeling and RV function

**FIGURE 6:**
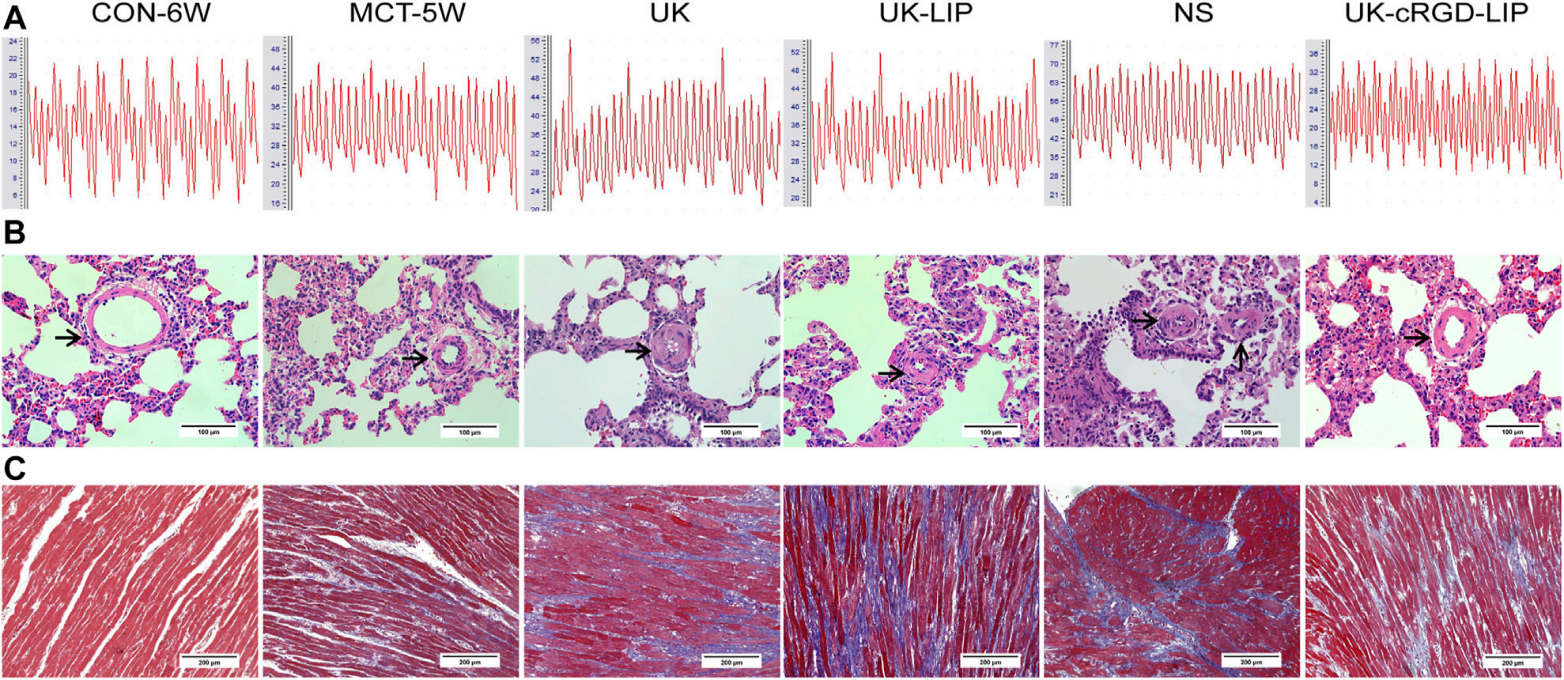
**(A)** Representative pulmonary artery pressure wavaforms obtained from CON-6W, MCT-5W, UK, UK-LIP, NS, and UK-cRGD-LIP rats. **(B)** Representative images (×400 original magnification) of H & E staining of lung tissues from CON-6W, MCT-5W, UK, UK-LIP, NS, and UK-cRGD-LIP rats. Black arrow points to pulmonary arteriole (20–100 μm diameter). **(C)** Representative images (×200 original magnification) of Masson’s trichrome staining of RV tissues from CON-6W, MCT-5W, UK, UK-LIP, NS and UK-cRGD-LIP rats. Red indicates muscle fibers; blue indicates collagen fibers.

**FIGURE 7:**
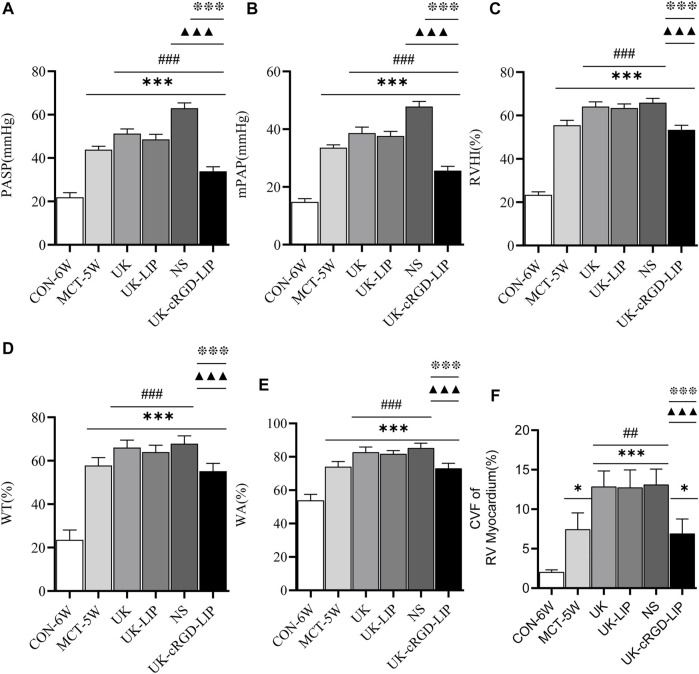
Reduction of *in-situ* thrombi in pulmonary arterioles alleviates the progression of MCT-induced PH in rats. **(A)** Pulmonary artery systolic pressure was measured in CON-6W, MCT-5W, UK, UK-LIP, NS, and UK-cRGD-LIP rats. **(B)** Mean pulmonary artery pressure was measured in CON-6W, MCT-5W, UK, UK-LIP, NS, and UK-cRGD-LIP rats. **(C)** Quantitative analysis of right ventricular hypertrophy index. **(D)** Quantitative analysis of vascular wall thickness. **(E)** Quantitative analysis of vascular wall area. **(F)** Quantitative analysis of fibrotic area. Values were presented as Mean ± SD. Data (CON-6W, *n* = 10; MCT-5W, *n* = 9; UK, *n* = 8; UK-LIP, *n* = 8; NS, *n* = 8; UK-cRGD-LIP, *n* = 9) were analyzed by one-way ANOVA **(A–E)** or Kruskal–Wallis test **(F)**. *p < 0.05 and ***p < 0.001 vs. CON-6W; ^**##**^ *p* < 0.01 and ^**###**^fn^###^ *p* < 0.001 vs. MCT-5W; ^▲▲▲^ *p* < 0.001 vs. UK or UK-LIP; ^❉❉❉^ *p* < 0.001 vs. NS.

To get a flavor of the role of pulmonary arteriole *in situ* thrombosis in PH, we observed pulmonary artery pressure as well as cardiac and pulmonary pathological tissues in MCT rats. PASP (Figure 6A and Figure 7A) and mPAP (Figure 6B and Figure 7B) were notably lower in rats in the UK-cRGD-LIP group compared with the MCT-5W, NS, UK, and UK-LIP groups. Compared with the UK-cRGD-LIP group, the results of RVHI (Figure 7C), WT (Figure 6B and Figure 7D), WA (Figure 6B and Figure 7E), and CVF of RV myocardium (Figure 6C and Figure 7F) suggested that RV myocardial remodeling and pulmonary vascular remodeling were further aggravated in the NS, UK, and UK-LIP groups. Surprisingly, RVHI, WT, WA, and CVF of RV myocardium in the MCT-5W group were not significantly different from those in the UK-cRGD-LIP group (Figures 7C–F). The above outcomes demonstrate that *in-situ* thrombi of pulmonary arterioles by targeted therapy can significantly reduce pulmonary arterial pressure and thus limit the pathological development of PH.

**FIGURE 8:**
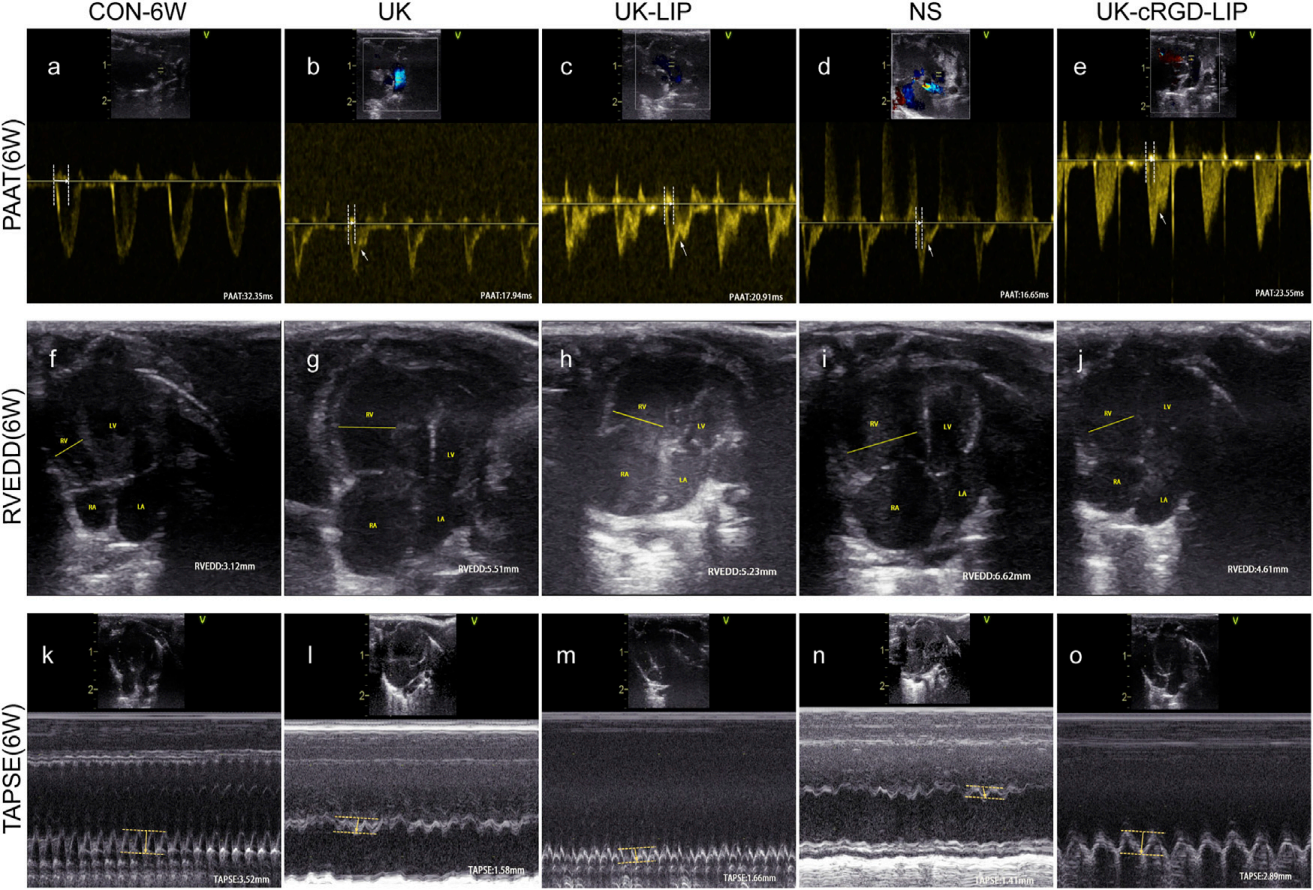
Representative echocardiography images of CON-6W, UK, UK-LIP, NS and UK-cRGD-LIP rats. **(A–E)** PAAT: Pulse-wave Doppler of views at the parasternal short-axis section. White arrow pointed to midsystolic notching. **(F–J)** RVEDD: Apical four-chamber view. Yellow line highlighted right ventricle internal diameter during diastole. **(K–O)** TAPSE: Tricuspid annular plane systolic excursion was recorded by M-mode echocardiography. Yellow arrow represented distance. Abbreviations: RV, right ventricular; LV, left ventricular; RA, right atrium; LA, left atrium.

**FIGURE 9:**
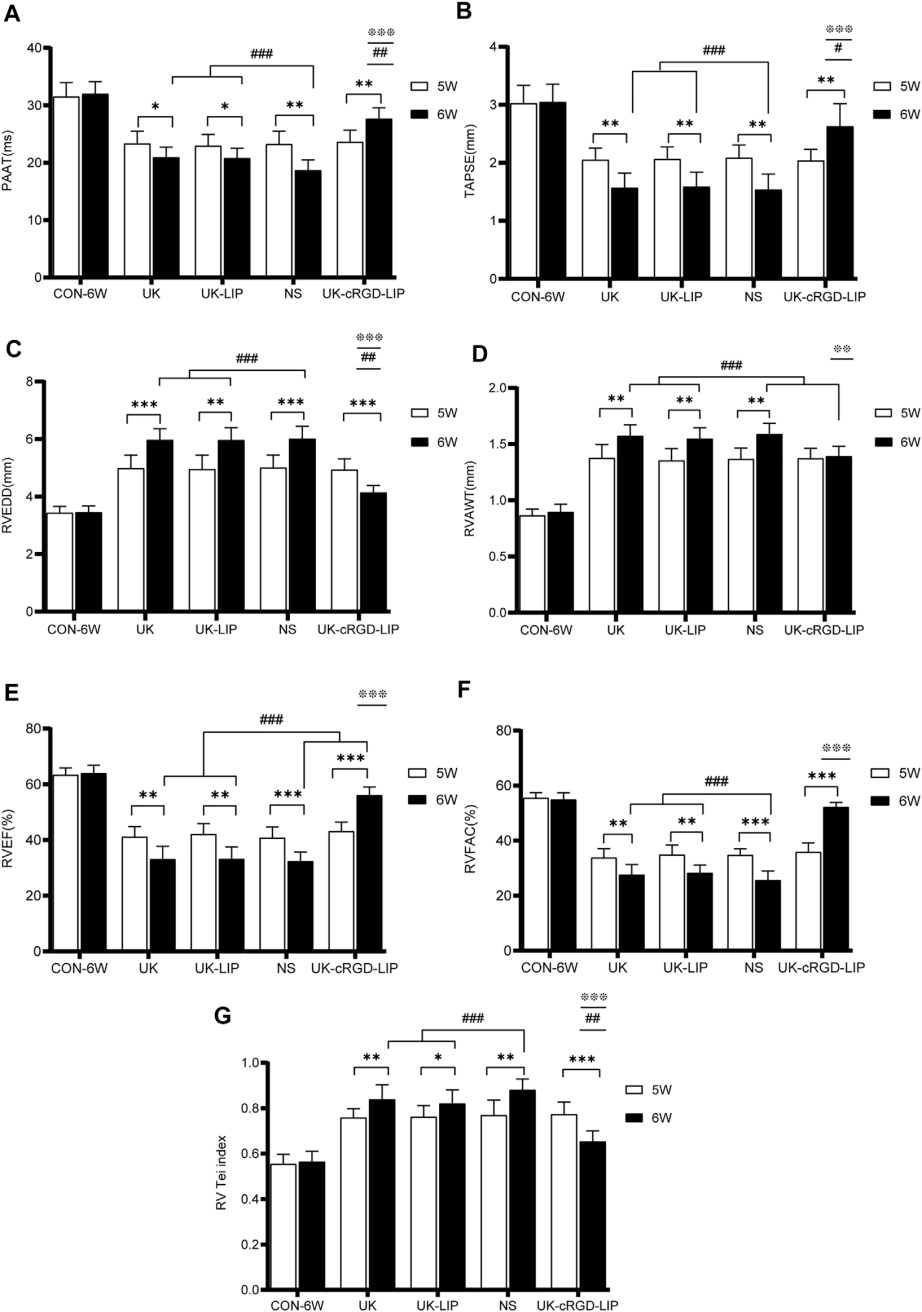
Echocardiography parameters of right ventricular function and morphology. **(A–G)** Analysis of PAAT, TAPSE, RVEDD, RVAWT, RVEF, RVFAC, RV Tei index from CON-6W, UK, UK-LIP, NS, and UK-cRGD-LIP rats. Values were presented as Mean ± SD. Data (CON-6W, *n* = 10; UK, *n* = 8; UK-LIP, *n* = 8; NS, *n* = 8; UK-cRGD-LIP, *n* = 9) were analyzed by unpaired Student’s t-test or one-way ANOVA. *p < 0.05, **p < 0.01, and ***p < 0.001. ^**#**^ *p* < 0.05, ^**##**^ *p* < 0.01, and ^**###**^ *p* < 0.001 vs. CON-6W (6W). ^❉^ *p* < 0.05, ^❉❉^ *p* < 0.01, and ^❉❉❉^ *p* < 0.001 vs. UK(6W), UK-LIP(6W), or NS(6W). Abbreviations: 5W, pre thrombolytic therapy; 6W, post thrombolytic therapy; PAAT, pulmonary artery acceleration time; RVEDD, right ventricular end-diastolic transverse diameter; TAPSE, tricuspid annular plane systolic excursion; RVEF, right ventricular ejection fraction; RVFAC, right ventricular area change fraction; RV Tei index, right ventricular myocardial work index; RVAWT, right ventricular anterior wall thickness.

To investigate whether targeted thrombolytic therapy for PH ameliorates RV function, we performed echocardiography in rats in the CON-6W and NS, UK, UK-LIP, and UK-cRGD-LIP groups pre and post thrombolytic treatment. In MCT rats, PAAT displayed mid-systolic notching, a characteristic echocardiographic feature of elevated pulmonary artery pressure (Figure 8 and Figure 9A). Compared with pre thrombolytic treatment, the rats in the NS, UK, and UK-LIP groups’ echocardiography values indicated further deterioration of right ventricular function and morphology post thrombolytic treatment (increased RVEDD; decreased TAPSE, RVEF, and RVFAC; ascended RV Tei index; thickened RVAWT) (Figure 8 and Figures 9B–G). In contrast, the RV systolic function was significantly improved in the UK-cRGD-LIP group post thrombolytic treatment. There was no significant difference in echocardiography values pre and post treatment in the CON-6W group. In the MCT-induced rat model of PH, RVEF <40%, RV FAC <35%, and TASPE<1.75 mm have been widely used as ultrasonographic value gauges for right heart failure (Mariano-Goulart et al., 2003; Hardziyenka et al., 2006; Rudski et al., 2010). RVEF (Figure 9E), RVFAC (Figure 9F), and TAPSE (Figure 8 and Figure 9B) in rats in the NS, UK, and UK-LIP groups reached above cut-off values post conventional thrombolytic treatment. These results suggest that effective targeted dissolution of pulmonary arterioles *in-situ* thrombi in PH can improve RV function markedly.

### *In vivo* safety evaluation

**FIGURE 10:**
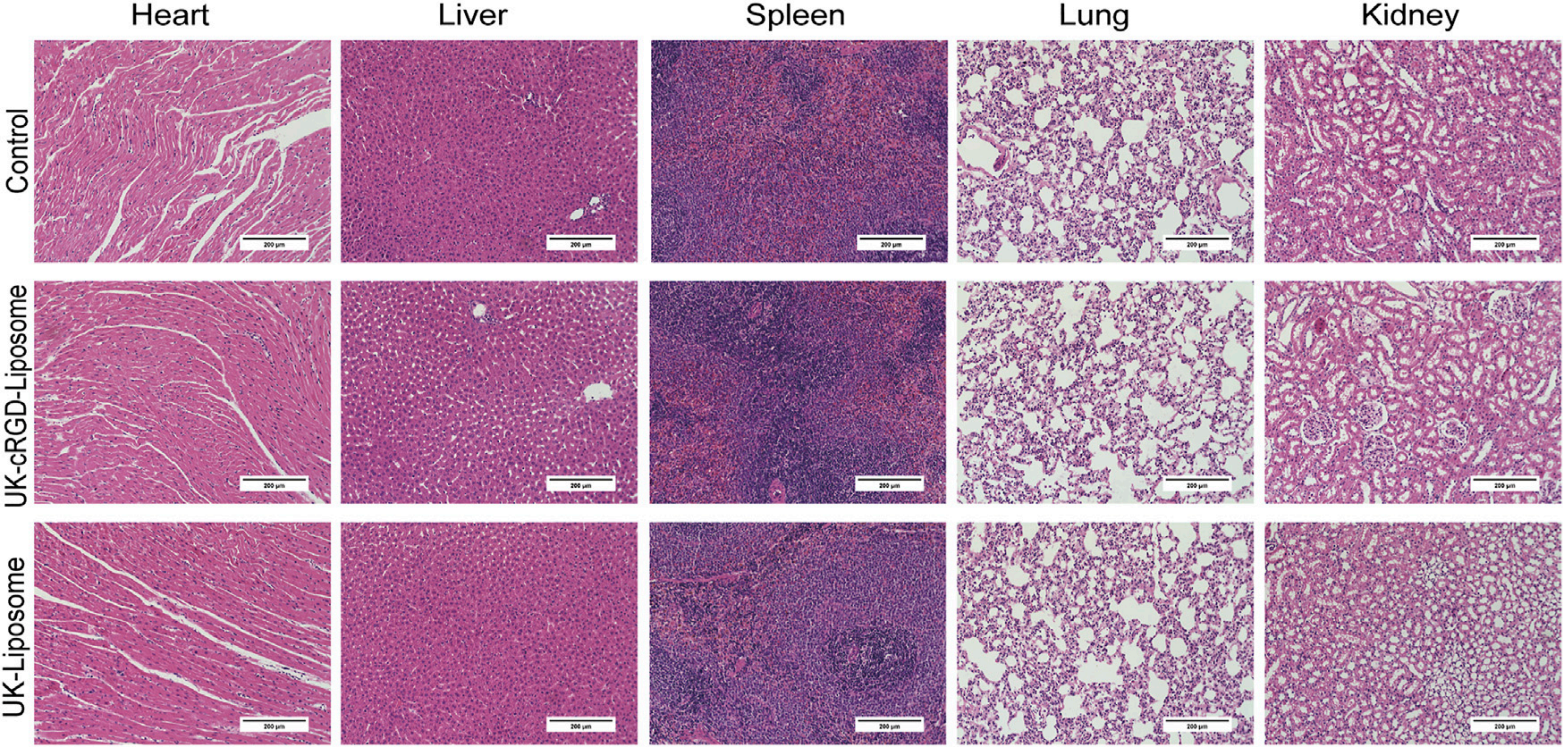
H & E staining of the major organs collected from healthy SD rats 1 week after tail vein injection of UK-cRGD-Liposome, UK-Liposome or normal saline (Control). Abbreviations: UK-cRGD-Liposome, urokinase-loaded cycle RGD-decorated liposome; UK-Liposome, urokinase-loaded liposome.

Since MCT rats could suffer some degree of damage to their major organs, healthy SD rats were reselected for histological examination to investigate the possible toxic effects of nanoscale drug carriers *in vivo*. During the treatment period, all groups of rats survived. In addition, no abnormal clinical behaviors or signs were observed in any of the groups. The H&E staining results of the heart, liver, spleen, lung, and kidney tissue sections showed that no significant injuries or pathophysiological changes were observed in the major organs of the rats in each group (Figure 10). Therefore, the cRGD-modified nanoscale active targeting drug delivery vehicle has good biocompatibility.

## Discussion

To the best of our knowledge, this is the first research to show that UK-cRGD-Liposome can target the dissolution of chronically formed pulmonary arterioles *in-situ* thrombi in PH. Furthermore, we also found that pulmonary arterioles *in-situ* thrombi in PH with targeted therapy can considerably enhance RV function and alleviate the progression of PH. These findings demonstrate that micro-thrombi formed *in-situ* in pulmonary arterioles play a critical role in the progression of PH.

### Animal models of PH

Currently, typical experimental PH models include the MCT-induced PH model, the chronic hypoxia-induced PH model, and the surgically established body-pulmonary circulation shunt-induced PH model (Wu et al., 2015; Kawai et al., 2022). MCT is a pyrrolizidine alkaloid extracted from the seeds of the Crotalaria spectabilis (Benoist et al., 2014). The MCT alkaloid selectively injures pulmonary vascular endothelial cells through activated monocrotaline pyrrole (MCTP) by cytochrome P450 in the liver, thereby inducing an increase in pulmonary artery pressure (Li et al., 2016). Fibrinogen-like protein two expressed in pulmonary arterioles in PH can directly activate the coagulation system and cause massive fibrin deposition, and the activated coagulation system and damaged vascular endothelial cells cause activated platelet aggregation. These processes result in *in-situ* thrombosis in pulmonary arterioles (Fan et al., 2019). Furthermore, neonate rat lungs exhibit alveolar and capillary surface development similar to that of humans (Zoetis and Hurtt, 2003). Therefore, this study selected the method of subcutaneous injection of MCT to establish an experimental PH model. In our study, the presence of *in situ* thrombi composed of deposited fibrin was observed in the pulmonary arterioles of MCT rats by fibrin staining (modified MSB method) (Figure 5), which not only further confirmed the previous study but also provided an experimental model for the thrombolytic study of UK-cRGD-Liposome in pulmonary arteriole *in-situ* thrombus.

### The efficacy of targeted thrombolysis in PH

The RGD peptide not only specifically binds the GPIIb/IIIa receptor on the surface of activated platelets but also inhibits the GPIIb/IIIa receptor from binding to fibrinogen, thus preventing platelet aggregation (Figure 1) (Pawlowski et al., 2017; Rima et al., 2018). Moreover, PEGylated liposomes could accumulate *in vivo via* enhanced permeability and retention (EPR) effect (Sercombe et al., 2015). Pharmacokinetic study of FITC-cRGD liposome showed that the plasma clearance half-life of cRGD liposome was 2.5 h, which was several times that of free urokinase. The tail bleeding assay in mice implied that the cRGD liposome could significantly reduce the bleeding side effects of urokinase (Zhang et al., 2018). Based on the Zhang et al. (2018) study, ourselves successfully prepared UK-cRGD-Liposome with smaller particle size (176.8 nm), higher encapsulation efficiency (58.83%), higher drug loading capacity (40.64%), and more stable release capacity (most of the urokinase could be released stably and continuously in first 8 h) (Rao, 2020). In this study, the results of the thrombolysis experimental demonstrated that UK-cRGD-Liposome was four times more efficient at thrombolysis than that of the same dose of urokinase (Table 1). These results not only demonstrate that UK-cRGD-Liposome can effectively deliver urokinase to chronically formed pulmonary arterioles *in-situ* thrombi but also further improve the basic research of cRGD peptide targeting activated platelets. Furthermore, liposomes have been widely used as nanoscale drug delivery vehicles in clinical settings, such as daunorubicin citrate liposomes and vincristine liposomes have been licensed for enhancing the efficacy of chemotherapeutics and for overcoming drug resistance by the US Food and Drug Administration (FDA) (Janko et al., 2019). The RGD sequence-based antiplatelet medication tirofiban also has been widely used in clinical (Suntravat et al., 2013). As a consequence, UK-cRGD-Liposome is safe and efficient nano-drug delivery systems that are expected to be novel targeted thrombolytic medications.

Guidelines for the diagnosis and treatment of pulmonary hypertension have classified PH into five groups on the basis of pathophysiology, etiology, and hemodynamics (Humbert et al., 2022). Group 1: Pulmonary arterial hypertension (PAH), including idiopathic PH, hereditary PH, and drug or toxin-induced PH. MCT-induced PH belongs to PAH (Group 1); in PAH patients, improvement in RV function was critical to lowering mortality (Potus et al., 2015; Clapham et al., 2020). PAH induces an increase in RV afterload, which eventually results in fibrosis of RV myocardial tissue and RV dysfunction when the afterload exceeds RV compensatory capacity (Vang et al., 2021). In our study, rats in the UK-cRGD-LIP group had considerably improved RV systolic function and RV pumping efficiency (Figures 9B,E–G) post targeted thrombolytic therapy. More notably, pulmonary artery pressure was reduced in the UK, UK-LIP, and UK-cRGD-LIP groups compared to the NS group. Meanwhile, pulmonary artery pressure in the UK-cRGD-LIP group was also significantly lower than that in the MCT-5W group (Figure 6A and Figures 7A,B). This finding supports the hypothesis that *in-situ* thrombus in PH can cause a significant increase in pulmonary artery pressure by increasing mechanical obstruction of the pulmonary vascular system, which can lead to further worsening in PH. Additionally, we also observed that CVF and WT, WA were no longer further elevated in the post targeted thrombolytic therapy (Figures 6B,C and Figures 7D–F). These results suggest that reducing *in-situ* thrombosis can significantly restrict the progression of pulmonary vascular remodeling and RV remodeling in PH. In summary, targeted thrombolytic therapy can clearly lower pulmonary artery pressure in PH, leading to improved right ventricle-pulmonary artery coupling and RV function.

### Limitation

In this study, albeit targeted thrombolytic therapy improved RV function, it failed to reverse pulmonary vascular remodeling and RV remodeling, which may be related to the short therapy time point and observation period. Hence, it is worthy of further study to grasp the therapy time point of pulmonary arteriole *in-situ* thrombus in pulmonary hypertension as well as to utilize UK-cRGD-Liposome more effectively and to improve the production process of UK-cRGD-Liposome.

## Conclusion

In conclusion, our study demonstrates that this biocompatible UK-cRGD-Liposome could targeted therapy the chronically formed pulmonary arterioles *in-situ* thrombi in PH. Furthermore, the most intriguing finding from our study is that reducing *in-situ* thrombosis of pulmonary arterioles can alleviate the progression of PH and improve RV function, which could be a beneficial exploration of the potential mechanisms of *in-situ* thrombus involved in the development of PH. In summary, UK-cRGD-Liposome offers a promising target drug-delivery system for thrombolytic therapy as well as a beneficial reference for the targeted therapy of *in-situ* thrombus in PH.

## Data Availability

All datasets presented in this study are included in the article, further inquiries can be directed to the corresponding author.

## References

[B1] Akbarzadeh A. Rezaei-Sadabady R. Davaran S. Joo S. W. Zarghami N. Hanifehpour Y. (2013). Liposome: Classification, preparation, and applications. Nanoscale Res. Lett. 8 (1), 102. 10.1186/1556-276x-8-102 23432972 PMC3599573

[B2] Altaf F. Wu S. Kasim V. (2021). Role of fibrinolytic enzymes in anti-thrombosis therapy. Front. Mol. Biosci. 8, 680397. 10.3389/fmolb.2021.680397 34124160 PMC8194080

[B3] Benoist D. Stones R. Benson A. P. Fowler E. D. Drinkhill M. J. Hardy M. E. (2014). Systems approach to the study of stretch and arrhythmias in right ventricular failure induced in rats by monocrotaline. Prog. Biophys. Mol. Biol. 115 (2-3), 162–172. 10.1016/j.pbiomolbio.2014.06.008 25016242 PMC4210667

[B4] Boucly A. Weatherald J. Savale L. Jaïs X. Cottin V. Prevot G. (2017). Risk assessment, prognosis and guideline implementation in pulmonary arterial hypertension. Eur. Respir. J. 50 (2), 1700889. 10.1183/13993003.00889-2017 28775050

[B5] Chen R. Ni S. Chen W. Liu M. Feng J. Hu K. (2021). Improved anti-triple negative breast cancer effects of docetaxel by RGD-modified lipid-core micelles. Int. J. Nanomedicine 16, 5265–5279. 10.2147/ijn.S313166 34376979 PMC8349197

[B6] Clapham K. R. Highland K. B. Rao Y. Fares W. H. (2020). Reduced RVSWI is associated with increased mortality in connective tissue disease associated pulmonary arterial hypertension. Front. Cardiovasc. Med. 7, 77. 10.3389/fcvm.2020.00077 32426373 PMC7203784

[B7] Cullivan S. Murphy C. A. Weiss L. Comer S. P. Kevane B. McCullagh B. (2021). Platelets, extracellular vesicles and coagulation in pulmonary arterial hypertension. Pulm. Circ. 11 (3), 1–9. 10.1177/20458940211021036 PMC8182202 34158919

[B8] Deng Y. Wu W. Guo S. Chen Y. Liu C. Gao X. (2017). Altered mTOR and Beclin-1 mediated autophagic activation during right ventricular remodeling in monocrotaline-induced pulmonary hypertension. Respir. Res. 18 (1), 53. 10.1186/s12931-017-0536-7 28340591 PMC5366117

[B9] Fan C. Wang J. Mao C. Li W. Liu K. Wang Z. (2019). The FGL2 prothrombinase contributes to the pathological process of experimental pulmonary hypertension. J. Appl. Physiol. (1985). 127 (6), 1677–1687. 10.1152/japplphysiol.00396.2019 31580221

[B10] Fernandez R. A. Sundivakkam P. Smith K. A. Zeifman A. S. Drennan A. R. Yuan J. X. (2012). Pathogenic role of store-operated and receptor-operated Ca2+ channels in pulmonary arterial hypertension. J. Signal Transduct. 2012, 1–16. 10.1155/2012/951497 PMC3465915 23056939

[B11] Garcia Ribeiro R. S. Belderbos S. Danhier P. Gallo J. Manshian B. B. Gallez B. (2019). Targeting tumor cells and neovascularization using RGD-functionalized magnetoliposomes. Int. J. Nanomedicine 14, 5911–5924. 10.2147/ijn.S214041 31534330 PMC6681073

[B12] Guo Y. Wu J. Jia H. Chen W. Shao C. Zhao L. (2015). Balancing the expression and production of a heterodimeric protein: Recombinant agkisacutacin as a novel antithrombotic drug candidate. Sci. Rep. 5, 11730. 10.1038/srep11730 26144864 PMC4491848

[B13] Guo Y. Wu W. Cen Z. Li X. Kong Q. Zhou Q. (2014). IL-22-producing Th22 cells play a protective role in CVB3-induced chronic myocarditis and dilated cardiomyopathy by inhibiting myocardial fibrosis. Virol. J. 11, 230. 10.1186/s12985-014-0230-z 25547181 PMC4304148

[B14] Hardziyenka M. Campian M. E. de Bruin-Bon H. A. Michel M. C. Tan H. L. (2006). Sequence of echocardiographic changes during development of right ventricular failure in rat. J. Am. Soc. Echocardiogr. 19 (10), 1272–1279. 10.1016/j.echo.2006.04.036 17000367

[B15] Hu Y. Yu D. Wang Z. Hou J. Tyagi R. Liang Y. (2019). Purification and characterization of a novel, highly potent fibrinolytic enzyme from Bacillus subtilis DC27 screened from Douchi, a traditional Chinese fermented soybean food. Sci. Rep. 9 (1), 9235. 10.1038/s41598-019-45686-y 31239529 PMC6592948

[B16] Huang G. Zhou Z. Srinivasan R. Penn M. S. Kottke-Marchant K. Marchant R. E. (2008). Affinity manipulation of surface-conjugated RGD peptide to modulate binding of liposomes to activated platelets. Biomaterials 29 (11), 1676–1685. 10.1016/j.biomaterials.2007.12.015 18192005 PMC2278119

[B17] Humbert M. Kovacs G. Hoeper M. M. Badagliacca R. Berger R. M. F. Brida M. (2022). 2022 ESC/ERS Guidelines for the diagnosis and treatment of pulmonary hypertension. Eur. Heart J. 00, 1–114. 10.1093/eurheartj/ehac237

[B18] Jalce G. Guignabert C. (2020). Multiple roles of macrophage migration inhibitory factor in pulmonary hypertension. Am. J. Physiology-Lung Cell. Mol. Physiology 318 (1), L1–l9. 10.1152/ajplung.00234.2019 31577159

[B19] Janko C. Ratschker T. Nguyen K. Zschiesche L. Tietze R. Lyer S. (2019). Functionalized superparamagnetic iron oxide nanoparticles (SPIONs) as platform for the targeted multimodal tumor therapy. Front. Oncol. 9, 59. 10.3389/fonc.2019.00059 30815389 PMC6382019

[B20] Kawai M. Zhang E. Kabwe J. C. Okada A. Maruyama J. Sawada H. (2022). Lung damage created by high tidal volume ventilation in rats with monocrotaline-induced pulmonary hypertension. BMC Pulm. Med. 22 (1), 78. 10.1186/s12890-022-01867-6 35247989 PMC8897872

[B21] Khan A. A. Allemailem K. S. Almatroodi S. A. Almatroudi A. Rahmani A. H. (2020). Recent strategies towards the surface modification of liposomes: An innovative approach for different clinical applications. 3 Biotech. 10 (4), 163. 10.1007/s13205-020-2144-3 PMC7062946 32206497

[B22] Koudelka S. Mikulik R. Mašek J. Raška M. Turánek Knotigová P. Miller A. D. (2016). Liposomal nanocarriers for plasminogen activators. J. Control. Release 227, 45–57. 10.1016/j.jconrel.2016.02.019 26876783

[B23] Li L. S. Luo Y. M. Liu J. Zhang Y. Fu X. X. Yang D. L. (2016). Icariin inhibits pulmonary hypertension induced by monocrotaline through enhancement of NO/cGMP signaling pathway in rats. Evidence-Based Complementary Altern. Med. 2016, 1–10. 10.1155/2016/7915415 PMC4904099 27366192

[B24] Liu H. Zhang R. Zhang D. Zhang C. Zhang Z. Fu X. (2022). Cyclic RGD-decorated liposomal gossypol AT-101 targeting for enhanced antitumor effect. Int. J. Nanomedicine 17, 227–244. 10.2147/ijn.S341824 35068931 PMC8766252

[B25] Liu M. Wu B. Wang W. Z. Lee L. M. Zhang S. H. Kong L. Z. (2007). Stroke in China: Epidemiology, prevention, and management strategies. Lancet Neurol. 6 (5), 456–464. 10.1016/s1474-4422(07)70004-2 17434100

[B26] Mariano-Goulart D. Eberlé M. C. Boudousq V. Hejazi-Moughari A. Piot C. Caderas de Kerleau C. (2003). Major increase in brain natriuretic peptide indicates right ventricular systolic dysfunction in patients with heart failure. Eur. J. Heart Fail. 5 (4), 481–488. 10.1016/s1388-9842(03)00041-2 12921809

[B27] Olschewski H. Rich S. (2018). Are anticoagulants still indicated in pulmonary arterial hypertension? Pulm. Circ. 8 (4), 2045894018807681–2045894018807685. 10.1177/2045894018807681 30284508 PMC6202749

[B28] Pan Q. Zhang J. Li X. Zou Q. Zhang P. Luo Y. (2019). Construction of novel multifunctional luminescent nanoparticles based on DNA bridging and their inhibitory effect on tumor growth. RSC Adv. 9 (26), 15042–15052. 10.1039/c9ra01381d 35516329 PMC9064234

[B29] Paulin R. Sutendra G. Gurtu V. Dromparis P. Haromy A. Provencher S. (2015). A miR-208-Mef2 axis drives the decompensation of right ventricular function in pulmonary hypertension. Circ. Res. 116 (1), 56–69. 10.1161/circresaha.115.303910 25287062

[B30] Pawlowski C. L. Li W. Sun M. Ravichandran K. Hickman D. Kos C. (2017). Platelet microparticle-inspired clot-responsive nanomedicine for targeted fibrinolysis. Biomaterials 128, 94–108. 10.1016/j.biomaterials.2017.03.012 28314136 PMC6526940

[B31] Potus F. Ruffenach G. Dahou A. Thebault C. Breuils-Bonnet S. Tremblay È. (2015). Downregulation of MicroRNA-126 contributes to the failing right ventricle in pulmonary arterial hypertension. Circulation 132 (10), 932–943. 10.1161/circulationaha.115.016382 26162916

[B32] Price L. C. Montani D. Tcherakian C. Dorfmüller P. Souza R. Gambaryan N. (2011). Dexamethasone reverses monocrotaline-induced pulmonary arterial hypertension in rats. Eur. Respir. J. 37 (4), 813–822. 10.1183/09031936.00028310 20693255

[B33] Rao H. Che X. Pan X. Huang G. Chen X. Wu J. (2021). A comparative study on physicochemical properties of two kinds of targeting thrombus urokinase-loaded microbubbles. Chongqing Med. 50 (05), 845–849. 10.3969/j.issn.1671-8348.2021.05.001

[B34] Rao H. (2020). Nanning, China: Guangxi Medical University. Master's Thesis. 10.27038/d.cnki.ggxyu.2020.000454 Experimental study on the preparation and physicochemical properties of urokinase-loaded cyclic RGD-modified liposomes

[B35] Rawal H. Suman A. Bhoite R. R. Kanwal A. Young R. K. Aronow W. S. (2021). Anticoagulation in pulmonary arterial hypertension: Do we know the answer? Curr. Probl. Cardiol. 46 (3), 100738. 10.1016/j.cpcardiol.2020.100738 33250263

[B36] Rima M. Alavi Naini S. M. Karam M. Sadek R. Sabatier J. M. Fajloun Z. (2018). Vipers of the Middle East: A Rich source of bioactive molecules. Molecules 23 (10), 2721. 10.3390/molecules23102721 PMC6222703 30360399

[B37] Rudski L. G. Lai W. W. Afilalo J. Hua L. Handschumacher M. D. Chandrasekaran K. (2010). Guidelines for the echocardiographic assessment of the right heart in adults: A report from the American society of echocardiography. J. Am. Soc. Echocardiogr. 23 (7), 685–713. quiz 786-688. 10.1016/j.echo.2010.05.010 20620859

[B38] Sercombe L. Veerati T. Moheimani F. Wu S. Y. Sood A. K. Hua S. (2015). Advances and challenges of liposome assisted drug delivery. Front. Pharmacol. 6, 286. 10.3389/fphar.2015.00286 26648870 PMC4664963

[B39] Sommer N. Ghofrani H. A. Pak O. Bonnet S. Provencher S. Sitbon O. (2021). Current and future treatments of pulmonary arterial hypertension. Br. J. Pharmacol. 178 (1), 6–30. 10.1111/bph.15016 32034759

[B40] Suntravat M. Jia Y. Lucena S. E. Sánchez E. E. Pérez J. C. (2013). cDNA cloning of a snake venom metalloproteinase from the eastern diamondback rattlesnake (*Crotalus adamanteus*), and the expression of its disintegrin domain with anti-platelet effects. Toxicon 64, 43–54. 10.1016/j.toxicon.2012.12.025 23313448 PMC3570744

[B41] Vaidya B. Agrawal G. P. Vyas S. P. (2012). Functionalized carriers for the improved delivery of plasminogen activators. Int. J. Pharm. X. 424 (1-2), 1–11. 10.1016/j.ijpharm.2011.12.032 22222184

[B42] Vang A. da Silva Gonçalves Bos D. Fernandez-Nicolas A. Zhang P. Morrison A. R. Mancini T. J. (2021). α7 Nicotinic acetylcholine receptor mediates right ventricular fibrosis and diastolic dysfunction in pulmonary hypertension. JCI Insight 6 (12), 142945. 10.1172/jci.insight.142945 33974567 PMC8262476

[B43] Velayati A. Valerio M. G. Shen M. Tariq S. Lanier G. M. Aronow W. S. (2016). Update on pulmonary arterial hypertension pharmacotherapy. Postgrad. Med. 128 (5), 460–473. 10.1080/00325481.2016.1188664 27232660

[B44] Vonk-Noordegraaf A. Haddad F. Chin K. M. Forfia P. R. Kawut S. M. Lumens J. (2013). Right heart adaptation to pulmonary arterial hypertension: Physiology and pathobiology. J. Am. Coll. Cardiol. 62 (25), D22–D33. 10.1016/j.jacc.2013.10.027 24355638

[B45] White R. J. Meoli D. F. Swarthout R. F. Kallop D. Y. Galaria II Harvey J. L. (2007). Plexiform-like lesions and increased tissue factor expression in a rat model of severe pulmonary arterial hypertension. Am. J. Physiology-Lung Cell. Mol. Physiology 293 (3), L583–L590. 10.1152/ajplung.00321.2006 17586694

[B46] Wu J. Luo X. Huang Y. He Y. Li Z. (2015). Hemodynamics and right-ventricle functional characteristics of a swine carotid artery-jugular vein shunt model of pulmonary arterial hypertension: An 18-month experimental study. Exp. Biol. Med. (Maywood). 240 (10), 1362–1372. 10.1177/1535370214566561 25595189 PMC4935261

[B47] Wu Y. Adi D. Long M. Wang J. Liu F. Gai M. T. (2016). 4-Phenylbutyric acid induces protection against pulmonary arterial hypertension in rats. PLoS One 11 (6), e0157538. 10.1371/journal.pone.0157538 27304885 PMC4909300

[B48] Yang P. S. Kim D. H. Lee Y. J. Lee S. E. Kang W. J. Chang H. J. (2014). Glycyrrhizin, inhibitor of high mobility group box-1, attenuates monocrotaline-induced pulmonary hypertension and vascular remodeling in rats. Respir. Res. 15, 148. 10.1186/s12931-014-0148-4 25420924 PMC4248446

[B49] Zhang N. Li C. Zhou D. Ding C. Jin Y. Tian Q. (2018). Cyclic RGD functionalized liposomes encapsulating urokinase for thrombolysis. Acta Biomater. 70, 227–236. 10.1016/j.actbio.2018.01.038 29412186

[B50] Zhou X. Wang Z. Xu B. Ji N. Meng P. Gu L. (2021). Long non-coding RNA NORAD protects against cerebral ischemia/reperfusion injury induced brain damage, cell apoptosis, oxidative stress and inflammation by regulating miR-30a-5p/YWHAG. Bioengineered 12 (2), 9174–9188. 10.1080/21655979.2021.1995115 34709972 PMC8810080

[B51] Zoetis T. Hurtt M. E. (2003). Species comparison of lung development. Birth Defect. Res. B 68 (2), 121–124. 10.1002/bdrb.10014 12866703

